# Hematuria Following Rapid Bladder Decompression

**DOI:** 10.5811/cpcem.2017.9.35803

**Published:** 2017-10-18

**Authors:** Christopher Gabriel, Jeffrey R. Suchard

**Affiliations:** * University of California, Irvine School of Medicine, Irvine, California; † University of California Irvine Medical Center, Department of Emergency Medicine, Orange, California

## CASE PRESENTATION

**Image 1:**
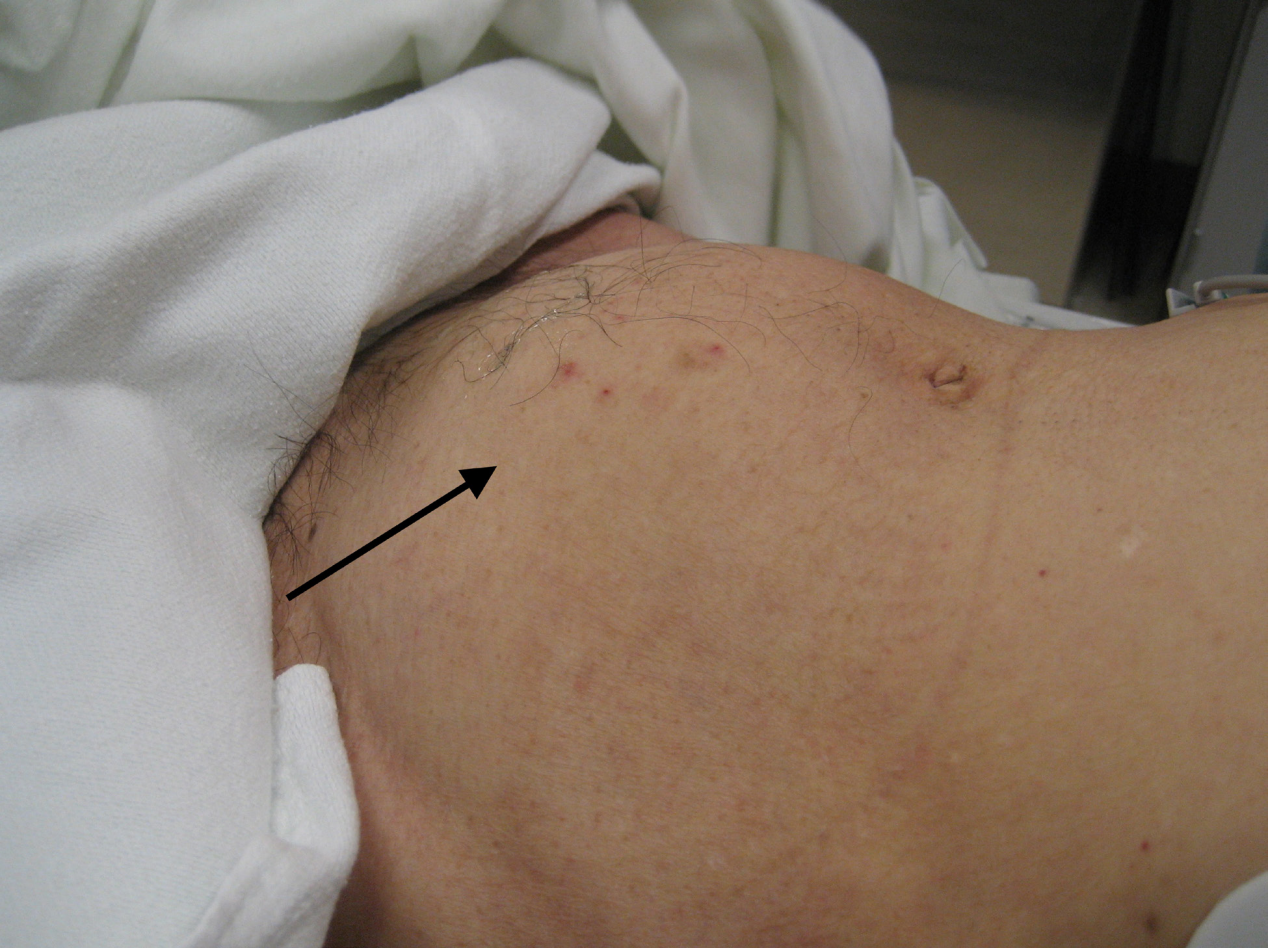
Visibly distended bladder prior to Foley catheter insertion.

**Image 2:**
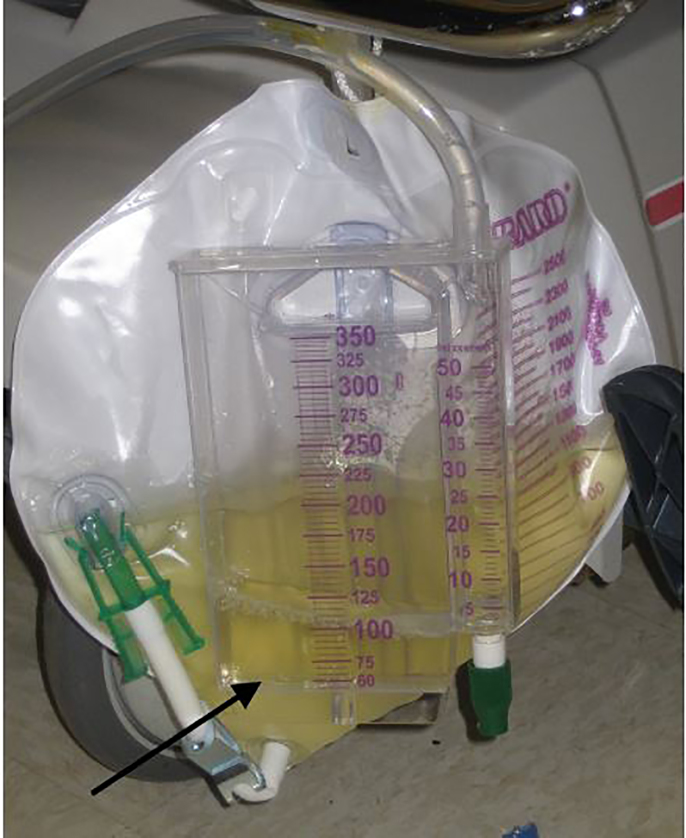
Initial drainage of the bladder demonstrated clear urine.

**Image 3:**
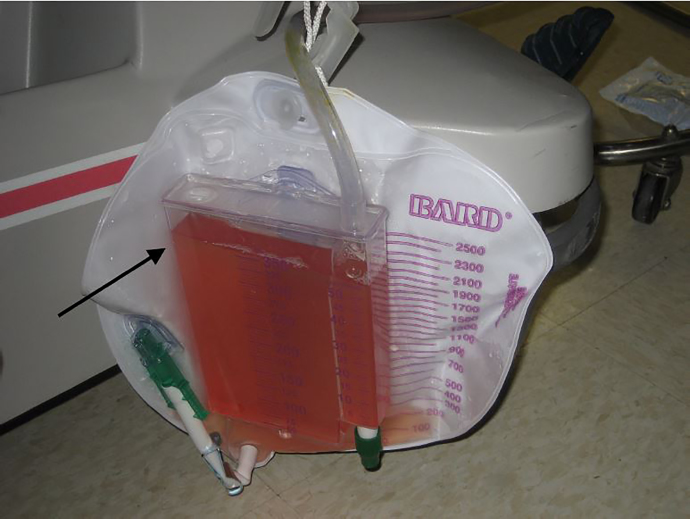
Gross hematuria seen three hours after rapid bladder decompression.

A 52-year-old man with prostatic hyperplasia presented to the emergency department with complaints of lower abdominal pain worsening over three days and inability to urinate. Abdominal examination revealed a protuberant, distended bladder (Image 1). A Foley catheter was inserted, with immediate return of clear urine (Image 2) and relief of the patient’s pain. Over two liters of urine were drained initially, and the urine collection bag was subsequently emptied. Three hours after catheterization, gross hematuria was evident (Image 3).

## DISCUSSION

Urinary outlet obstruction is commonly treated by insertion of a Foley catheter, allowing drainage of the distended bladder. Previous recommendations have suggested gradual drainage of an obstructed bladder, for instance by intermittent catheter clamping, to avoid complications of hematuria, hypotension, and post-obstructive diuresis, although the value of this practice is debatable.^1^,^2^ Hematuria occurs in 2–16% of patients following quick, complete relief of urinary obstruction.^1^ A randomized, controlled study of 294 patients found no significant difference in the incidence of hematuria following rapid vs. gradual bladder emptying (10.5% and 11.3% respectively).^2^ Even when hematuria occurs following bladder decompression, it is typically benign and self-limited.

A systematic literature review of related studies published from 1920 to 1997 found no cases of hematuria severe enough to necessitate further invasive therapy, such as bladder irrigation or blood transfusion.^1^ However, a 2012 case report details a rare patient with severe hematuria following rapid bladder decompression.^3^ The hematuria resulted in worsening anemia (hemoglobin decreased from 9.5 to 7.8 g/dL) and oliguria due to bilateral ureteral thrombus formation; this patient was transfused blood and underwent bladder irrigation, cystoscopy, and percutaneous nephrostomy. Although complications of rapid bladder decompression can occur very rarely, evidence from literature reviews and controlled trials supports rapid and complete emptying of the obstructed urinary bladder.^1^,^2^

**CPC-EM Capsule**

What do we already know about this clinical entity?

Traditional warnings against rapid bladder decompression may prolong definitive care in the ED and result in use of additional resources.

What is the major impact of the image(s)?

Hematuria is demonstrated after bladder decompression, although the patient suffered no adverse complications.

How might this improve emergency medicine practice?

Despite the possibility of hematuria, ED patients with bladder outlet obstruction can be rapidly decompressed with low risk of serious sequelae.
